# Plasmid mediated colistin resistant *mcr*-1 and co-existence of *OXA*-48 among *Escherichia coli* from clinical and poultry isolates: first report from Nepal

**DOI:** 10.1186/s13099-020-00382-5

**Published:** 2020-09-17

**Authors:** Bijaya Muktan, Upendra Thapa Shrestha, Binod Dhungel, Bagish Chandra Mishra, Nabaraj Shrestha, Nabaraj Adhikari, Megha Raj Banjara, Bipin Adhikari, Komal Raj Rijal, Prakash Ghimire

**Affiliations:** 1 Central Department of Microbiology, Tribhuvan University, Kirtipur, Kathmandu, Nepal; 2 Kantipur Hospital Pvt. Ltd., Tinkune, Kathmandu, Nepal; 3 Central Veterinary Laboratory, Ministry of Agriculture, Land Management and Cooperatives, Government of Nepal, Tripureshwor, Kathmandu, Nepal; 4 Centre for Tropical Medicine and Global Health, Nuffield Department of Medicine, University of Oxford, Oxford, UK

**Keywords:** *mcr*-1, *OXA*-48, Colistin resistant *E. coli*, MDR, Polymerase chain reaction

## Abstract

**Background:**

Plasmid-mediated resistance to the last-resort drugs: carbapenems and colistin is an emerging public health threat. The studies on the prevalence and co-expression of resistant genes among livestock and human pathogens are rare in Nepal. This is the first study in Nepal exploring the prevalence and co-existence of colistin resistance gene, *mcr*-1 along with carbapenemase resistance gene, *OXA*-48 in *Escherichia coli* isolated from poultry and clinical specimens.

**Methods:**

A total of 240 rectal swabs from chickens of five different poultry farms of Kathmandu valley and 705 mid-stream urine samples from human subjects attending Kantipur Hospital, Kathmandu were collected between August, 2018 and March, 2019. Rectal swabs and urine specimens were cultured. *E. coli* isolated from the specimens were screened for antimicrobial susceptibility testing (AST) using disk diffusion method’. Minimum inhibitory concentration (MIC) of colistin was determined by agar dilution method using 0.5 µg/ml to 32 µg/ml. The *E. coli* isolates were first screened for *mcr*-*1* followed by screening for *OXA*-*48* genes using conventional Polymerase chain reaction (PCR).

**Results:**

Of the total samples analyzed, *E. coli* was isolated from 31.7% (76/240) of poultry and 7.9% (56/705) of clinical specimens. In AST, 80% (61/76) of *E. coli* from poultry and 79% (44/56) from clinical specimens were MDR. The phenotypic prevalence of colistin resistance in poultry specimens were 31.6% (24/76) and clinical specimens were 21.4% (12/56). In PCR assay, 27.6% (21/76) of poultry and 19.6% (11/56) of clinical isolates had colistin resistant *mcr*-*1* gene. MICs value of *E. coli* isolates ranged from 4 to 32 (µg/ml) in both clinical and poultry isolates. Prevalence of co-existing carbapenem resistance gene, *OXA*-48, among colistin resistant *mcr*-1 positive isolates was 38% (8/21) in poultry specimens and 18.2% (2/11) in clinical specimens.

**Conclusions:**

The high prevalence of colistin and carbapenem resistant genes, and their co-existence in plasmid DNA of *E. coli* isolates in this study suggests the possible spread to other animal, human and environmental pathogens. Molecular methods in addition to the conventional diagnostics in laboratories can help in early diagnosis, effective management and control of their potential transmission.

## Background

The plasmid-encoded colistin resistant gene, *mcr*-1 was first reported in *E. coli* isolates from livestock and human specimens in China [1]. Until the discovery of *mcr*-1, all reported polymyxin resistance mechanisms were chromosomally mediated, due to mutation and regulatory changes [2], and had never been reported to occur via horizontal gene transfer [3]. Two earlier mechanisms: natural and phenotypic mechanisms were suggested among colistin resistant strains, the former occurring via mutations of bacterial genomes while the latter was the result of adaptive mechanism [3]. A number of *mcr*-1 strains have been reported worldwide among several species of *Enterobacteriaceae* in a short span of time since its first report in 2016 [4].

*mcr*-*1* gene is not only limited to *E. coli* but also has been spreading among other members of *Enterobacteriaceae* co-existing with other resistance genes [5]. Several retrospective studies performed worldwide showed that *mcr*-1 had been circulating undetected for at least 20 years [6]. Subsequent findings of 11 new genetic variant of *mcr*-1 across different countries show increasing divergence of colistin resistance mechanism [7]. There is a strong association of ISApl1 genomic insertion site and *mcr*-1, which causes demographic expansion and global distribution [2]. Furthermore, insertion of *mcr*-1 into *E. coli* chromosomes may enable it to become intrinsically resistant, which is expected to become more prevalent in future [8].

*mcr*-1 genes carrying colistin resistant *E. coli* isolates are challenging ‘one health’ concept as these strains have been isolated from humans, animals, and environ-ments from all continents [9]. Finding of *mcr*-1 gene carrying bacteria in natural environments shows possibility of transference of *mcr*-1 carrying *Enterobacteriaceae* to humans via food chain [10].

There is a growing number of findings reporting the rapid surge of carbapenemase including *blaVIM*-1, *blaNDM*-1 and *OXA*-48 among carbapenem resistance *Enterobacteriaceae* (CRE) in human beings [1, 11, 12], though the CRE is still rare in animals [13]. The emergence and global spread of co-existing carbapenem resistance with plasmid-mediated colistin resistance (*mcr*-1) in gram-negative bacterial pathogens, particularly among the members of *Enterobacteriaceae* can be catastrophic [13, 14]. There is an increasing concern on the co-existence of colistin and carbapenem resistances in *Enterobacteriaceae* from human and animal samples, because this combination can limit the therapeutic options in the treatment of MDR bacteria [4, 15].

In Nepal, there is a pervasive use of antibiotics in food animals farming as growth promoters in addition to indiscriminate over the counter use among humans [16, 17]. The colistin resistance can increase in exponential proportion because of its widespread use in food animals and human beings and has been supported by the detection of *mcr*-1 gene from environment, food, animals and human beings [18]. Although global reports on colistin resistance in human and environment are increasing [19], there is only one previous report till date from Nepal. Recent studies reported from Nepal showed 26.66% of colistin-resistant *E. coli* harbored *mcr*-1 gene isolated from the chicken meats [20] and 33.3% of carbapenem resistant *E*. *coli* harbored OXA-48 isolated from urine samples [21]. The main objective of this study was to explore the prevalence and co-existence of colistin resistance gene, *mcr*-1 along with carbapenemase resistance gene, *OXA*-48 in *Escherichia coli* isolated from poultry farms located in Kathmandu, Kavrepalanchok and Bhaktapur and human specimens (urine) from patients attending Kantipur Hospital, Tinkune, Kathmandu between August, 2018 and March, 2019.

## Methods

### Study design and study sample

This cross-sectional study was carried out between August 2018 and March 2019 in Bhaktapur Kavrepalanchok, and Kathmandu districts. The clinical specimens were collected from Kantipur Hospital, Tinkune Kathmandu. The study subjects included patients of all ages and both gender (male and female) attending the hospital with suspicion of UTI and all of the participants provided written informed consent to participate in the study. A total of 705 mid-stream urine samples from patients (male, N = 315 and female, N = 390) suspected of urinary tract infection (UTI) were collected in a sterile, clean, well-labeled, and leak proof container with no visible signs of contamination and were transported to laboratory [22].

The poultry specimens were collected from selected poultry farms in Kavrepalanchok (Panauti), Bhaktapur (Darjeeling Height, Duwakot), Kathmandu (Sundarijal and Dhakal Gau). A total of 240 rectal swabs of chicken were collected and inoculated into buffered peptone water, kept in insulated ice-cold box and were transported to laboratory within 1 hour.

### Culture of specimens and identification of the isolates

The samples were observed for macroscopic, microscopic and culture characteristics. Urine samples were inoculated on Cysteine Lactose Electrolyte Deficient (CLED) (Hi Media, India) agar using a standard calibrated loop (4 mm). The collected rectal swabs with inoculated tubes were incubated for 18–24 h at 37 °C, and were sub-cultured on MacConkey agar (MA) (Hi Media, India). Inoculated CLED media plates were incubated aerobically at 37 °C for 18–24 h. Thus, obtained colony growth of gram-negative rods suspected of *E. coli* were further sub-cultured aerobically on Nutrient Agar (NA) from both MA and CLED media plates. *E. coli* from both specimens was identified on the basis of colony morphology, staining, biochemical tests and a greenish-metallic sheen of colonies formed on Eosin Methylene Blue (EMB) agar [23, 24].

## Antibiotic susceptibility test (AST)

All isolates of *E. coli* were tested for antibiotic susceptibility using modified Kirby Bauer disc diffusion method based on the Clinical and Laboratory Standard Institute guidelines [25]. Isolates were tested for resistance against amoxiclave (30 µg), ceftazidime (30 µg), cefixime (5 µg), ciprofloxacin (5 µg), gentamicin (10 µg), imipenem (10 µg), meropenem (10 µg), piperacillin/tazobactam (100/10 µg) and tetracycline (30 µg) (HiMedia, India). Results were interpreted as sensitive, intermediate and resistant [25]. Isolates showing non-susceptibility (either resistance or intermediate) to at least one antimicrobial agent in three or more of the categories were considered as multi-drug resistant (MDR) [26].

### Screening of colistin resistant *E. coli*

In AST, minimum inhibitory concentration (MIC) for colistin was determined by agar and broth micro-dilution methods using 0.5 µg/ml to 32 µg/ml. *E. coli* isolates showing visible growth on colistin concentration of 2 µg/ml or more than 2 µg/ml were detected as colistin resistant isolates [25].

### Extraction of plasmid DNA and PCR amplification of colistin resistance gene (mcr-1)

The plasmid DNA was extracted using alkaline lysis method. The extracted plasmids were then suspended in TE buffer, labeled well and stored at – 20 °C [27]. *mcr*-1 gene of plasmid carried by *E. coli* isolates was amplified using primer pairs CLR5-F (5ʹCGGTCAGTCCGTTTGTTC-3ʹ) and CLR5-R (5ʹ-CTTGGTCGGTCTGTA GGG-3ʹ) as forward and reverse primers [1]. Reaction volume was set as 25 µl by adding 21 µl of 1X master mix, 0.5 µl each of forward and reverse primer and 3 µl of DNA template. The optimized PCR amplification of *mcr*-1 gene is 95 °C for 15 min to activate hot-star; 30 cycle of denaturation at 94 °C for 30 s; annealing at 57 °C for 1:30 min; extension at 72º for 1:30 min, and final extension at 72 °C for 10 min and holding at 4 °C for 10 min.

### PCR amplification of carbapenem resistance gene (OXA-48)

*OXA*-48 gene responsible for the resistance of carbapenem drug was amplified using primer pairs as forward sequence *OXA*-48_forward primers (FP) (5′GCTTGATCGCCCTCGATT-3′) and reverse sequence *OXA*-48_reverse primers (RP) (3′GATTTGCTCCGTGGCCGAAA-5′). Reaction volume was set as 25 µl by adding 21 µl of 1× master mix, 0.5 µl each of forward and reverse primer and 3 µl of DNA template. The optimized PCR amplification of *OXA*-48 gene is 95 °C for 15 min to activate the hotstar, 30 cycle of denaturation at 94 °C for 30 s, annealing at 57 °C for 1:30 min and extension at 72º for 1:30 min and final extension at 72 °C for 10 min and holding at 4 °C for 10 min [28].

### Agarose gel electrophoresis

The agarose gel electrophoresis of extracted plasmid DNA and amplified PCR product was performed, followed by confirmation of *mcr*-1 and *OXA*-48 gene by visualizing in UV transillumination [28].

## Results

### Distribution of samples and prevalence of *E. coli* in poultry and clinical specimens

**Fig. 1:**
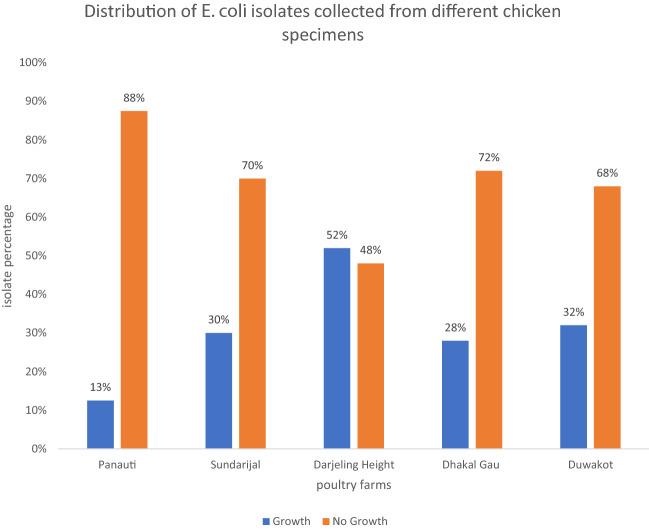
Farm wise growth distribution of *E. coli* isolates

The distribution of poultry samples according to farms with previous history of vaccination, colistin antibiotic use, number of samples collected, number of *E. coli* isolates and the presence of colistin resistant isolates are illustrated in Table 1. The growth pattern observed in samples from each farm is presented in Fig. 1. Overall, the prevalence of *E*. *coli* in poultry specimens was (31.7%;76/240) whereas it was (7.9%; 56/705) in clinical specimens.

**Table 1:** Farm wise epidemiological data of colistin resistant *E. coli*

District	Poultry sites	Number of samples taken	Vaccination	Use of colistin	*E. coli* isolated	Colistin resistant *E. coli* isolated
Kavrepalanchok	Panauti	40	+	–	5	2
Bhaktapur	Duwakot	50	+	–	16	9
	Darjeling Height	50	+	+	26	6
Kathmandu	Sundarijal	50	+	+	15	2
	Dhakal Gau	50	+	+	14	2
Total		240			76	21

Among 705 urine specimens, (18.4%; 130/705) showed bacterial growth. Of 130 bacterial growth, (43%;56/130) were *E. coli*. On age wise distribution of UTI patients, 44.6% (58/130) were from age group (16–45) years, (32.3%;42/130) were from age group > 45 years and (23.1%; 30/130) were from age group (0–15) years respectively.

### Antibiotic susceptibility test (AST) of E. coli isolates

Among the total of 76 *E. coli* isolates from poultry, highest resistance was found in tetracycline (67.1%; 51/76) followed by amoxiclave (55.3%; 42/76), ciprofloxacin (50%;38/76), nalidixic acid (50%;38/76) and imipenem (32.9%;25/76). Similarly, among the total of 56 *E. coli* isolates from clinical specimen, highest number of isolates were resistant to cefixime (71.6%;40/56) followed by ceftazidime (66.1%;37/56), ciprofloxacin (53.6%;30/56) and piperacillin/ tazobactam (39.3%;22/56) (Table 2).

**Table 2:** Antibiotic resistance profile of *E. coli* isolated from poultry and clinical specimens

Antibiotics	Resistance (%)	
	Poultry (n = 76)	Clinical (n = 56)
Amoxiclave (30 µg)	42 (55.3)	3 (5.4)
Ceftazidime (30µ g)	4 (5.3)	37 (66.1)
Cefixime (5 µg)	10 (13.2)	40 (71.4)
Ciprofloxacin (5 µg)	38 (50)	30 (53.6)
Gentamicin (10 µg)	21 (27.6)	12 (21.4)
Imipenem (10 µg)	25 (32.9)	2 (3.6)
Meropenem (10 µg)	2 (2.6)	0
Nalidixic acid (30 µg)	38 (50)	11 (19.6)
Piperacillin/Tazobactam (100/10 µg)	0	22 (39.3)
Tetracycline (30 µg)	51 (67.1)	21 (37.5)

**Fig. 2:**
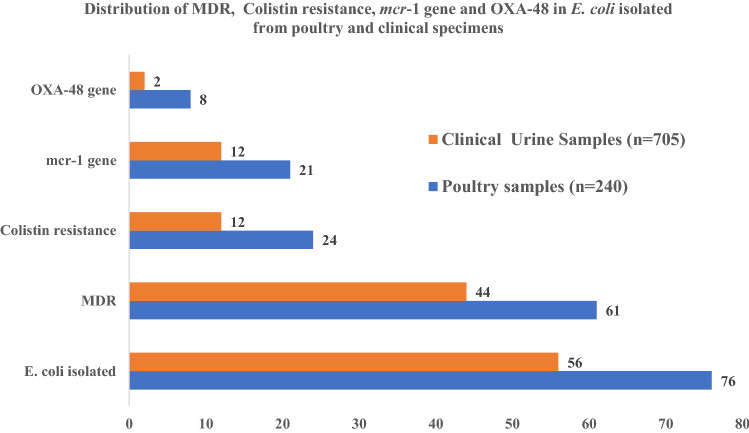
Distribution of MDR, colistin resistance, mcr-1 gene and OXA-48 in *E. coli*

In AST, (80%; 61/76) of poultry specimens and (79%; 44/56) of clinical specimens were MDR (Fig. 2).

### Antimicrobial resistance phenotypes of clinical and poultry E. coli isolates

Antibiotic resistance pattern of MDR *E. coli* isolates were determined by 12 different antibiotic resistance phenotypes (R- phenotypes). Among them the most resistant pattern in poultry *E. coli* isolates were imipenem-tetracycline-amoxiclave (21%; 16/76) followed by imipenem-ciprofloxacin-amoxiclav (19.7%; 15/76) and ciprofloxacin- tetracycline-nalidixic (18.4%; 14/76). Among clinical *E. coli* isolates, the most resistant pattern was ceftazidime-cefixime-tetracycline (21.4%; 12/56) followed by cefixime-nalidixic acid-tetracycline (8.9%; 5/56) (Table 3).

**Table 3:** Antibiotic resistance phenotypes of *E. coli* isolated from poultry and clinical specimens

S.N	Antimicrobial resistance pattern	Poultry *E. coli* Resistant isolates N (%)	Clinical *E. coli* Resistant isolates N (%)
1	CIP/TE/NA	14 (18.4)	2 (3.6%)
2	CIP/GEN/AMC	9 (11.8)	
3	IMP/TE/AMC	16 (21)	
4	IMP/CIP/AMC	15 (19.7)	
5	CFM/NA/TE	3 (4)	5 (8.9%)
6	GEN/NA/AMC	10 (13.2)	
7	IMP/CAZ/CIP	1 (1.3)	1 (1.8%)
8	CAZ/CFM/TE	2 (2.6)	12 (21.4%)
9	GEN/IMP/CIP	3 (4)	1 (1.8%)
10	CIP/TE/GEN/AMC	4 (5.2)	
11	TE/AMC/GEN/NA	6 (8)	
12	CIP/TE/AMC/NA	8 (10.5)	

*AMC* Amoxiclave, *CAZ* Ceftazidime, *CFM* Cefixime, *CIP* Ciprofloxacin, *GEN* Gentamicin, *IMP* Imipenem, *NA* Nalidixic acid, *TE* Tetracycline

### Determination of MIC and colistin resistant *E. coli* isolates

MICs value of *E. coli* isolates ranged from 4 to 32 (µg/ml) in both clinical and poultry *E. coli* isolates (Table 4). In this assay, 31.6% (24/76) of poultry isolates and 21.43% (12/76) of clinical isolates were confirmed as colistin resistant (Fig. 2) (Table 5).

**Table 4:** MIC colistin resistant *E. coli* isolates of poultry and clinical specimens

S. No	Colistin concentration (MIC)	Resistant isolates		P -value
		Poultry (n = 24)	Clinical (n = 12)	
1	4 (µg/ml)	4 (16.7)	3 (25)	0.8
2	8 (µg/ml)	5 (20.8)	2 (16.7)	
3	16 (µg/ml)	7 (29.2)	4 (33.3)	
4	32 (µg/ml)	8 (33.3)	3 (25)

**Table 5:** Distribution of colistin resistance, *mcr*-*1* gene and OXA-48 gene among *E. coli* isolates of poultry and clinical specimens

Colistin resistant *E. coli* isolates		*mcr*-1 gene among colistin resistant *E. coli* isolates		OXA-48 gene positive isolates in *mcr*-1 gene positive *E. coli*	
Poultry (n = 76)	Clinical (n = 56)	Poultry (n = 24)	Clinical (n = 12)	Poultry (n = 21)	Clinical (n = 11)
24 (31.6%)	12 (21.4%)	21 (87.5%)	11 (91.6%)	8 (38%)	2 (18.2%)

### Prevalence of mcr-1 gene among colistin resistant clinical and poultry *E. coli* isolates

**Fig. 3:**
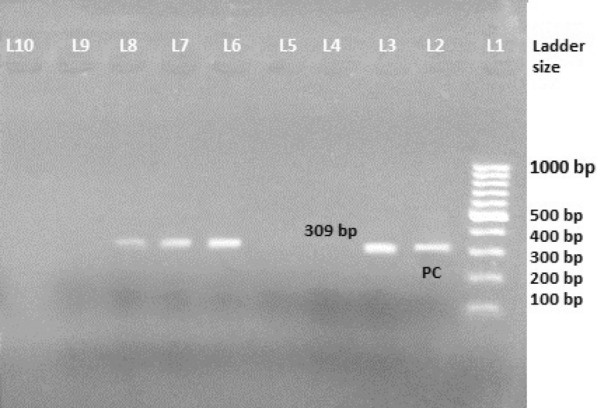
*mcr*-*1* gene in colistin resistant *E. coli* (Lane1, marker DNA (100–1000 bp), Lane L2 (positive control), L3, L6, L7 and L8 *mcr*-*1* positive (309 bp)

All *E. coli* isolates were screened for plasmid mediated *mcr*-1 gene using conventional PCR. In PCR assay, colistin resistance was found to be 27.6% in poultry isolates and 19.6% in clinical isolates. Among phenotypic colistin resistance, 87.5% (21/24) *E. coli* isolates from poultry specimens were tested positive for *mcr*-1 gene and 91.6% (11/12) of the clinical isolates (*E. coli*) were tested positive for *mcr*-1 gene (Table 5). The *mcr*-1 with 309 bp size is presented in Fig. 3.

### OXA-48 gene among *mcr*-1 positive colistin resistant *E. coli* isolates

**Fig. 4:**
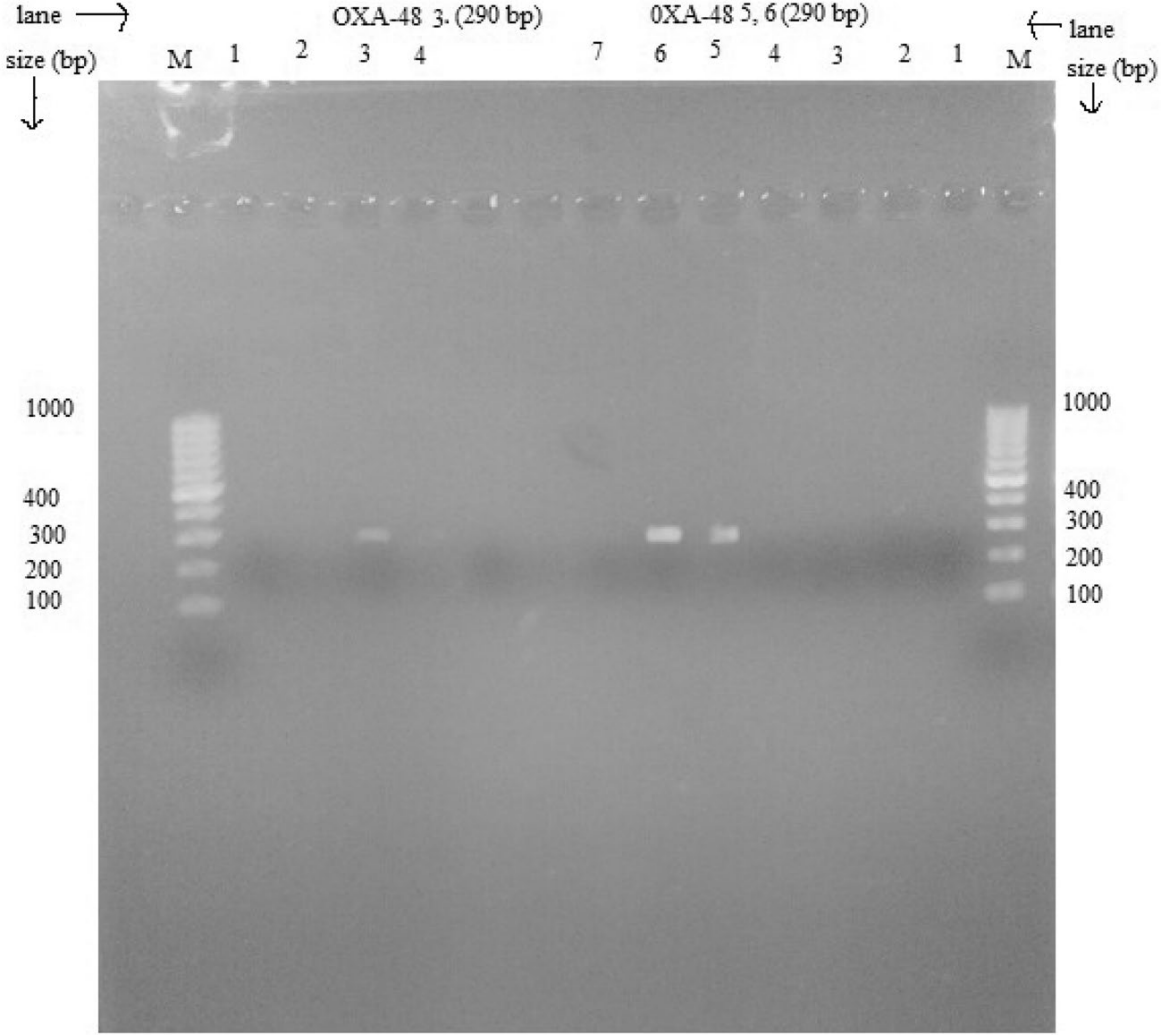
OXA-48 gene among mcr-1 positive *E. coli* isolates (Lane M, marker DNA (100–1000 bp), lane 5, 6 and 3, *OXA*-*48* gene (290 bp)

In this assay, 38% (8/21) of poultry isolates and 18.2% (2/11) of clinical isolates had carbapenem resistant *OXA*-48 gene (Table 5). The amplified *OXA*-48 gene with 290 bp is illustrated in Fig. 4.

## Discussion

This is the first report of plasmid mediated colistin resistance *mcr*-1 gene and *OXA*-48 *gene among mcr*-*1 positive colistin resistant E. coli isolates* from clinical and poultry specimens of Nepal. In this study, using PCR assay, colistin resistance was found to be 27.6% in poultry isolates and 19.6% in clinical isolates. The emergence and spread of CRE has obliged clinicians to use colistin—a drug notorious for its toxicity [29]. Colistin is the last resort drugs for these resistant isolates [30]. Increasingly over the recent years, morbidity, mortality and the economic consequences associated with colistin resistance are reported [31]. *mc*r-1 recently identified as resistance and transferable gene has been recovered from healthy carriers, food, environmental sources and clinical isolates [14]. Most of the *mcr*-1 positive strains have been proven to be carrying different carbapenemase genes such as *blaNDM*-9, *blaNDM*-5, *blaVIM*-1, *blaOXA*-48-type, and *blaKPC*- 2 [32]. This combination further limits the therapeutic options because both last resort drugs: colistin and carbapenems are challenged by this phenomenon [4]. Our study also confirmed the substantial presence of *mcr*-1 and *blaOXA*-48 among isolates of human and animal (poultry) origin.

In our study, 32% *E. coli* were isolated from poultry specimens consistent with a previous study that reported 36.4% growth of *E. coli* from poultry fed with colistin in Central Nepal [20]. Research conducted in China showed higher (80%) growth of *E. coli* from rectal swab of food animals [33]. The increased and variable rate of colistin resistance may be due to quality, concentration and extensive use of colistin in the livestock farming [34]. In clinical samples, *E. coli* remains as the most frequently isolated organisms which is consistent with several previous studies from Nepal [35–38]. The higher load of *E. coli* in urine samples may be due to their main role in urinary tract infections [35].

AST of poultry isolates showed more than 50% of resistance to most of the antibiotics used in this study. Similar resistance pattern was screened in China [2]. Another study reported from Iran had showed 77.7% resistance to ciprofloxacin, 33.3% resistance to gentamicin but no resistance to Imipenem [39]. A study from China reported higher rate of resistance to tetracycline (90%), cefixime (71.4%), and ceftazidime (66.1%) in *E. coli* isolates [33]. Another study from Pakistan reported higher resistance to gentamicin (77%) and ciprofloxacin (65%) in clinical isolates of *Enterobacteriaceae* [40]. Higher (79%) prevalence of multi-drug resistant isolates in our study is consistent with previous studies from Nepal [21, 41] and Pakistan [40]. The higher load of MDR isolates may be due to significant antibiotic pressure in the environment, irrational dose regimens, use in food animals and transmission of resistant isolates between people, animal and the environment [40]. In our study, 80% (61 out of 76) of *E. coli* from poultry were found to be MDR. Our finding was consistent with a study from Bharatpur, Nepal that showed MDR rate as 79.6% in poultry meat [42]. However, similar study from Bangladesh reported 100% MDR in *E. coli* isolates [43]. Open access between poultry farms and communities, unhygienic practice, inappropriate use of antibiotics, are some of the reasons attributed for emerging resistance patterns including MDRs in Nepal [33, 43, 44].

In this study, MIC value of colistin was observed up to 32 mg/L and found similar (4–32 µg/ml) dose in both poultry and clinical specimen. Most of the isolates of clinical specimen showed MIC value of 16 μg/ml while those *E. coli* isolates from poultry showed 32 μg/ml. Studies from Vietnam have reported lower MIC value of clinical isolates as 4 (µg/ml) [45] and MICs range of 4 to 16 μg/ml in *E. coli* isolates were reported from Chinese University Hospital [46]. The greater MIC value revealed these *E. coli* isolates as non-wild type [47]. The presence of multiple systems of resistance and multiple copy number of plasmids carrying *mcr*-1 gene may have played role in increasing the MIC value of colistin [48]. A multi-country study has reported consistent findings with our study [49]. A previous study from Germany has confirmed a presence of four plasmid carrying *mcr*-1 gene within *E. coli* isolates from patients, which indicated the role of *mcr*-1 gene for increased MIC of colistin [15].

In this study, the true occurrence of *mcr*-1 gene in poultry isolates is 27.6%. Similar result was reported by a study in Iran [50] while a study from Bangladesh reported a higher prevalence (94%) of colistin resistance in ESBLs *E. coli* isolates in poultry [51]. In this study, among 12 colistin resistant isolates, 11 (91.6%) were *mcr*-1 positive. The prevalence of *mcr*-1 gene is 19.6%. The findings of our study were in line with a study from Italy (8.3% resistance to colistin in hospital surfaces) [52]. The findings of our study, however differed from studies reported from China (low level of colistin resistance) [5], Denmark (higher rate of colistin resistance and presence of 87.5% *mcr*-1 gene among colistin resistant *Enterobacteriaceae*) [50], and Korea (14.3% colistin resistance) [9]. Nonetheless, these reports have shown the increasing resistance trend globally.

In this study, 38% and 18% of *mcr*-1 positive isolates from poultry and human specimens respectively harbored *OXA*-48 genes. This scenario is increasing in Asia and other continent in *E. coli* isolates [53, 54]. Most of the carbapenem resistance reports are concerned about CRE in clinical settings while this study reports CRE among *mcr*-1 positive isolates in clinical setting and poultry as well [55]. The presence of both *OXA*-48 and *mcr*-1 gene within a clinical *E. coli* isolates carried by plasmid is serious threat to public health. This study meanwhile could not predict whether both types were carried by the same plasmid.

## Strengths and limitations

This is the first study from Nepal that investigated the co-existence of *mcr*-1 and *OXA*-48. Since poultry remains one of the major sources of food in Nepal, use of antibiotics and its concurrent contribution in development of Antimicrobial Resistance (AMR) warrants an urgent attention to embrace ‘one health approach’ in Nepal. The findings of this study will be a fundamental reference for policy makers and clinicians to be informed about the characteristics and prevalence of colistin resistance which can subsequently guide the optimal treatment, use of antibiotics and infection control.

While our data clearly showed the presence of plasmid mediated *mcr*-1 and *OXA*-48 in both settings: poultry and humans, the findings could have been strengthened by conducting surveillance targeted at regional and national level for a whole picture for Nepal. Lack of whole genome sequencing in this study could not confirm the origin and transferability of the genes from animals to humans or vice-versa. Further studies using multi-locus sequence typing could be useful for epidemiological investigations.

## Conclusion

One fourth and one fifth of the plasmid mediated colistin resistance genes in *E. coli* from poultry specimens and clinical specimens indicate high burden of colistin resistance in Nepal. Furthermore, co-existence of colistin and carbapenem resistant genes; and their co-existence in plasmid DNA of *E. coli* isolates in this study suggests the possible spread to other animal, human and environmental pathogens. Molecular methods can aid in early diagnosis, effective management and potential control of transmission. One health approach is critical to fight against MDR that may have been cross-contaminated from environment, animal food and human beings.

## Abbreviations

AST: Antimicrobial susceptibility test
CAMPS: Cationic antimicrobial cyclic polypeptide
CPE: Cytopathic effect
CRE: Carbapenem resistant *E. coli*
CLSI: Clinical laboratory standard institute
ESBL: Extended spectrum β-lactamase
EUCAST: European committee on antimicrobial susceptibility testing
GNB: Gram negative bacteria
ICU: Intensive care unit
kDa: Kilo-Dalton
LPS: Lipopolysaccharide
MCR: Mediated colistin resistance
MDR: Multi-drug resistance
MIC: Minimum inhibitory concentration
ORF: Open reading frame
PCR: Polymerase chain reaction
UTI: Urinary tract infection
WHO: World health organization
XDR: Extensive/xeno-drug resistance
